# T2‐low severe asthma clinical spectrum and impact: The Greek PHOLLOW cross‐sectional study

**DOI:** 10.1002/clt2.70035

**Published:** 2025-01-29

**Authors:** Konstantinos Porpodis, Nikolaos Zias, Konstantinos Kostikas, Argyris Tzouvelekis, Michael Makris, George N. Konstantinou, Eleftherios Zervas, Stelios Loukides, Paschalis Steiropoulos, Konstantinos Katsoulis, Anastasios Palamidas, Aikaterini Syrigou, Maria Gangadi, Antonios Christopoulos, Dimosthenis Papapetrou, Fotios Psarros, Konstantinos Gourgoulianis, Eleni Tzortzaki, Stylianos K. Vittorakis, Ioannis Paraskevopoulos, Ilias Papanikolaou, Georgios Krommidas, Dimitrios Latsios, Nikolaos Tzanakis, Miltiadis Markatos, Angeliki Damianaki, Argyrios Manikas, Alexia Chatzipetrou, Dimitrios Vourdas, Ioanna Tsiouprou, Christina Papista, Marina Bartsakoulia, Nikolas Mathioudakis, Petros Galanakis, Petros Bakakos

**Affiliations:** ^1^ Pulmonary Department Aristotle University of Thessaloniki G. Papanikolaou Hospital Thessaloniki Greece; ^2^ Respiratory Department Navy Hospital of Athens Athens Greece; ^3^ Respiratory Medicine Department School of Medicine University of Ioannina Ioannina Greece; ^4^ Department of Respiratory Medicine Medical School University of Patras Patras Greece; ^5^ 2nd Department of Dermatology and Venereology Allergy Unit Medical School Attikon University General Hospital National and Kapodistrian University of Athens Athens Greece; ^6^ Department of Allergy and Clinical Immunology 424 General Military Training Hospital Thessaloniki Greece; ^7^ 7th Respiratory Department Athens Chest Hospital Sotiria Athens Greece; ^8^ 2nd Respiratory Department Medical School Attikon University Hospital National and Kapodistrian University of Athens Athens Greece; ^9^ Department of Respiratory Medicine Medical School University General Hospital Democritus University of Thrace Alexandroupolis Greece; ^10^ Pulmonary Department 424 Army General Hospital Thessaloniki Greece; ^11^ Athens Medical Center‐Marousi Clinic Athens Greece; ^12^ Allergy Department Sotiria General Hospital Athens Greece; ^13^ 10th Department of Pulmonary Medicine Athens Chest Hospital Sotiria Athens Greece; ^14^ Department of Respiratory Medicine University Hospital Patras Patra Greece; ^15^ Athens Medical Group Paleo Faliro Clinic Athens Greece; ^16^ Allergy Department Athens Naval Hospital Athens Greece; ^17^ Faculty of Medicine Department of Respiratory Medicine University Hospital of Larissa University of Thessaly Larissa Greece; ^18^ Outpatient Respiratory Clinic Heraklion Greece; ^19^ Private Practice Chania Greece; ^20^ 401 General Military Hospital Athens Athens Greece; ^21^ Pulmonary Department Corfu General Hospital Corfu Greece; ^22^ Private Practice Athens Greece; ^23^ Private Practice Drama Greece; ^24^ Department of Respiratory Medicine Medical School University General Hospital of Heraklion Laboratory of Molecular and Cellular Pneumonology University of Crete Heraklion Greece; ^25^ Outpatient Clinic for Pulmonary Diseases Chania Greece; ^26^ Pulmonary and Sleep Medical Department Chania General Hospital Agios Georgios Chania Greece; ^27^ European Interbalkan Medical Center Thessaloniki Greece; ^28^ 2nd Department of Dermatology and Venereology Allergy Unit University General Hospital Attikon National and Kapodistrian University of Athens Athens Greece; ^29^ Department of Allergy and Clinical Immunology 251 General Airforce Hospital Athens Greece; ^30^ Medical Affairs Department, Respiratory and Immunology AstraZeneca Athens Greece; ^31^ 1st University Department of Respiratory Medicine National and Kapodistrian University of Athens Athens Greece

**Keywords:** clinically significant exacerbations, quality of life, real‐world, symptom control, treatment patterns

## Abstract

**Background:**

Data on type 2 (T2)‐low severe asthma (SA) frequency is scarce, resulting in an undefined unmet therapeutic need in this patient population. Our objective was to assess the frequency and characterize the profile and burden of T2‐low SA in Greece.

**Methods:**

PHOLLOW was a cross‐sectional study of adult SA patients. Based on a novel proposed classification system, patients were classified as T2‐low if blood eosinophil count (BEC; cells/μL) was <150, fractional exhaled nitric oxide (FeNO) < 25 ppb and any allergy status or BEC < 150/FeNO < 50 ppb/no allergy or BEC < 300/FeNO < 25 ppb/no allergy. For patients receiving biologics and/or oral corticosteroids, only those with BEC < 150/FeNO < 25 ppb/no allergy/no response to therapy were classified as T2‐low. Secondary outcome measures were: Asthma Control Test (ACT^TM^), Mini‐Asthma Quality of Life Questionnaire (Mini‐AQLQ), hospital anxiety and depression scale (HADS), and Work Productivity and Activity Impairment:Respiratory Symptoms (WPAI:RS) questionnaire.

**Results:**

From 22‐Mar‐2022 to 15‐Mar‐2023, 602 eligible SA patients were enrolled. The frequency of T2‐low asthma was 20.1%. Of those, 71.1% had experienced ≥1 clinically significant exacerbations in the past year, 62.8% had ACT score <20 (uncontrolled asthma), and 22.3% were biologic‐treated. Mini‐AQLQ score was <6 (impairment) in 79.5% of patients, HADS‐total score was ≥15 (clinically significant emotional distress) in 43.8%, while median percent activity impairment and work productivity loss were 30.0 for both domains. Clinical and patient‐reported outcomes were worse among patients with ACT‐defined uncontrolled asthma.

**Conclusions:**

One‐fifth of SA patients present with a T2‐low endotype. These patients frequently have uncontrolled disease and experience impairments in their quality of life, emotions and work ability.

## 1 INTRODUCTION

Asthma affects more than 260 million people worldwide,^1^ with a lifetime self‐reported prevalence of 9.1% in Greece.^2^ About 4%–10% of asthmatics have severe asthma (SA), defined by the American Thoracic Society/European Respiratory Society as “asthma requiring treatment with high‐dose inhaled corticosteroids (ICS) plus a second controller and/or systemic corticosteroids to prevent it from becoming ‘uncontrolled’ or that remains ‘uncontrolled’ despite this therapy.”^3^, ^4^, ^5^, ^6^, ^7^ Patients with SA suffer from impaired health‐related quality of life (HRQoL) and exacerbations that may require emergency department (ED) visits and/or hospitalization, contributing to increased healthcare resource utilization (HCRU).^8^, ^9^

Two major endotypes of SA have been widely recognized, namely type‐2 (T2)‐high and T2‐low, depending on the type of airway inflammation.^10^ T2 inflammation is characterized by eosinophilic airway infiltration and overexpression of T2 cytokines, including interleukins (IL)‐4/5/13. T2‐high asthma can be allergic or non‐allergic and is usually characterized by high levels of blood eosinophil count (BEC) and fractional exhaled nitric oxide (FeNO) or sensitization to common aeroallergens independently of BEC/FENO. Conversely, T2‐low asthma is predominantly a neutrophilic or pauci‐granulocytic phenotype, characterized by the absence of T2‐high biomarkers, and lack of response to corticosteroids.^10^ Nevertheless, there are no T2‐low universally validated biomarkers in clinical practice.^10^, ^11^ As a result, there is a wide variation in the T2‐low classification schemes utilized across published studies and the reported frequency of T2‐low asthma among them ranges 9%–50%.^11^, ^12^, ^13^, ^14^, ^15^, ^16^, ^17^, ^18^, ^19^, ^20^, ^21^, ^22^, ^23^, ^24^, ^25^

Following the approval of the first biologic agent in SA management nearly 20 years ago, [omalizumab targeting immunoglobulin E (IgE)], multiple biologics targeting T2 cytokine signaling have been introduced.^26^ However, patients with T2‐high asthma are more likely to respond to these than patients with low T2‐inflammation markers.^27^, ^28^, ^29^, ^30^, ^31^ Efforts to identify targeted therapeutic approaches for T2‐low asthma are continuing.^11^, ^32^ This recognized unmet need led to the recent approval of Tezepelumab (an anti‐alarmin antibody blocking the activity of the thymic stromal lymphopoietin‐TSLP), which is a first‐in‐class drug and the only biologic approved in the European Union as add‐on maintenance treatment for SA without phenotype/biomarker limitations.^33^, ^34^ These advances together with optimized endotype‐definition tools can aid in delivering tailored therapeutic approaches and improving outcomes, particularly for the T2‐low SA subpopulation.

The available epidemiological data on asthma in Greece is limited to the general population (i.e., not specific to the severe condition).^2^, ^35^, ^36^, ^37^ In light of the rapidly evolving SA treatment landscape, the PHOLLOW study aimed to assess the T2‐low frequency and T2‐high endotypes among SA patients managed in routine care settings in Greece using a new well‐defined endotype classification system, taking into consideration patients on biologic and/or maintenance oral corticosteroid (mOCS) treatment.^38^ In addition, the study sought to describe the profile, treatments and burden of T2‐low SA patients in order to better inform future healthcare policy decisions.

## 2 METHODS

### 2.1 Study design

This cross‐section and retrospective chart review 2‐part study included outpatients with SA treated by asthma specialists (pulmonologists and allergists) in routine care settings in Greece. Details of the protocol and study design have been published elsewhere.^38^ In brief, we devised a new composite SA endotype classification system to categorize patients into T2‐low and T2‐high based on airway inflammation biomarkers (BEC and FeNO) and allergic/atopic status. For patients currently on biologic and/or mOCS treatment, the scoring system employed lower thresholds for T2‐inflammation detection (since such therapy can mask the T2 signature), while response to treatment was also factored in (since T2‐low asthma typically does not respond to these therapies).

Specifically, scores of 0/1/2 were assigned for BEC <150/150–299/ ≥ 300 cells/μL and FeNO <25/25–49/ ≥ 50 ppb, scores of 0/1 for negative/positive allergic (or atopic) status, and score of 0/2 for negative/positive response to biologic/mOCS therapy. Based on the cumulative score of the four aforementioned criteria (resulting from the multiple combinations of thresholds), two different definitions, namely BASE and STRICT, were utilized to classify patients into “Definite T2‐low,” “Possible T2‐low,” “Possible T2‐high,” and “Definite T2‐high”. Detailed information on the scoring system and SA endotype classification based on the two study definitions is shown in Table S1 and Figure S1. In summary, the following combinations of the four classification criteria were utilized to categorize patients as having T2‐low SA:

Possible/definite T2‐low based on BASE classification (total of 6 combinations):

Patients NOT treated with biologic and/or mOCS (4 combinations):

1. BEC < 150/FeNO < 50 ppb/any allergy status;
2. BEC < 150/any FeNO/no allergy;
3. BEC < 300/FeNO < 25 ppb/any allergy status;
4. BEC < 300/FeNO < 50 ppb/no allergy.

Patients treated with biologic and/or mOCS (2 combinations):

1. BEC < 150/FeNO < 25 ppb/any allergy status/no response to therapy;
2. BEC < 150/FeNO < 50 ppb/no allergy/no response to therapy.

Possible/definite T2‐low based on STRICT classification (total of 4 combinations):

Patients NOT treated with biologic and/or mOCS (3 combinations):

1. BEC < 150/FeNO < 25 ppb/any allergy status;
2. BEC < 150/FeNO < 50 ppb/no allergy;
3. BEC < 300/FeNO < 25 ppb/no allergy.

Patients treated with biologic and/or mOCS (1 combination):

1. BEC < 150/FeNO < 25 ppb/no allergy/no response to therapy.

Using the Asthma Control Test (ACT^TM^)^39^ patients with T2‐low SA were further stratified into “controlled” (ACT score ≥20) and “uncontrolled” (ACT score <20), based on asthma symptom control level at the study visit.

In part A of the study, top‐level patient data were collected from all eligible patients to assess frequency and predictors of T2‐low SA. Patients classified as T2‐low proceeded to part B of the study, where further characteristics and patient‐reported outcomes (PROs) were collected (Figure S1).

### 2.2 Study population

Eligibility criteria have been described elsewhere (refer to the publication of the protocol and study design).^38^ Briefly, the study enrolled adult patients with SA, physician‐diagnosed asthma for ≥12 months, and ≥2 BEC/ ≥ 1 IgE/ ≥ 1 FeNO available measurements within the previous 12 months before (and including) the study visit (≥1 IgE measurement before omalizumab initiation for omalizumab‐treated patients), who provided their consent to participate.

### 2.3 Ethical considerations

The study was designed and conducted in accordance with the ethical principles outlined in the Helsinki Declaration, the Guidelines for Good Pharmacoepidemiology Practice of the International Society for Pharmacoepidemiology, the STROBE (Strengthening the Reporting of Observational Studies in Epidemiology) recommendations where applicable, the EU General Data Protection Regulation, and local rules and regulations.

In accordance with current local regulatory requirements, the study protocol and patient's Informed Consent Form (ICF) were reviewed and approved by the competent site‐specific Institutional Review Boards of the participating hospitals before the enrollment of any patient and the performance of any study‐related procedures. Approval by a central Ethics Committee is not applicable to non‐interventional studies in Greece. All participants signed an ICF.

### 2.4 Study outcomes

The primary outcome was to assess the frequency of the BASE‐defined T2‐low asthma endotype among SA patients. Secondary outcomes presented herein included assessment of (i) the frequency of STRICT‐defined T2‐low asthma endotype, (ii) the level of asthma symptom control in the T2‐low subpopulation, and (iii) the following outcomes at the study visit in the T2‐low subpopulation and its subgroups with ACT‐defined “controlled” and “uncontrolled” asthma: characterization of the patient demographic/clinical profile; management strategies; clinically significant asthma exacerbations (CSEs) and asthma‐related HCRU over the past 12 months; spirometry‐based lung function; and PROs of Mini‐Asthma Quality of Life Questionnaire (Mini‐AQLQ), hospital anxiety and depression scale (HADS), and Work Productivity and Activity Impairment:Respiratory Symptoms (WPAI:RS) questionnaire. The exploratory objectives presented herein include the identification of factors influencing the frequency of T2‐low SA and the ACT‐defined asthma symptom control in the T2‐low SA subpopulation.^38^

### 2.5 Statistical analyses

A precision‐based sample size calculation was employed, resulting in a total planned sample of 600 patients and an anticipated size of ≥100 patients for the T2‐low subpopulation,^38^ thus ensuring a margin of error ≤10% for study outcomes (for more details refer to the publication of the protocol and study design).^38^

Continuous and categorical variables are displayed using descriptive statistics, that is, frequencies (%, *n*), mean, standard deviation, median, interquartile range (25th percentile—75th percentile). The Clopper–Pearson 95% confidence intervals (CI) were estimated for binomial proportions and the 95% Poisson CI for incidence rates. The association of factors of interest with binary outcomes (1. T2‐low SA phenotype “Yes” vs. “No”; 2. “ACT ≥ 20 Controlled” vs. “ACT < 20 Uncontrolled” in the T2‐low SA population) was evaluated through univariable and multivariable logistic regression models. Explanatory variables that caused complete/quasi separation and/or extremely unbalanced groups were not included in the analysis. Odds ratios (OR), 95% CIs and *p*‐values are presented.

To derive the most parsimonious multivariable model, a stepwise method based on minimization of Akaike information criterion (AIC) was used. The goodness of fit for the logistic regression models was assessed using the Hosmer‐Lemeshow test.

In addition, to identify whether “type of institution” (academic vs. other) and “administrative region” of the study site (Attica vs. outside Attica) were confounders of the aforementioned relationships, multivariable regression analysis was conducted. Evidence of confounding was defined as >10% change in the adjusted ORs compared to crude ORs (in either direction) with a concomitant change in statistical significance in the observed relationship between the examined factor and the outcome. In case a variable was identified as a confounder, it was to be directly included in the final multivariable model, that is, the model after the stepwise procedure. Furthermore, variables that were examined in both continuous and categorical form by univariable regression models were included in the multivariable model in the form corresponding to a lower *p*‐value in the univariable analysis.

Except for partial dates, no missing data imputation was applied. All statistical tests were two‐sided with 0.05 significance level. All analyses were performed using SAS v9.4 (SAS Institute, Cary, NC).

## 3 RESULTS

### 3.1 Patient disposition

Over a 12‐month recruitment period from 22 March 2022 (first patient in) to 15 March 2023 (last patient in), 613 patients were consecutively enrolled in 30 study sites. Eleven patients did not meet all eligibility criteria; thus, the analysis included 602 patients, 74.4% of whom were enrolled by hospital clinics, and 61.0% were treated with biologics and/or mOCS at enrollment.

### 3.2 T2‐low SA frequency

**FIGURE 1:**
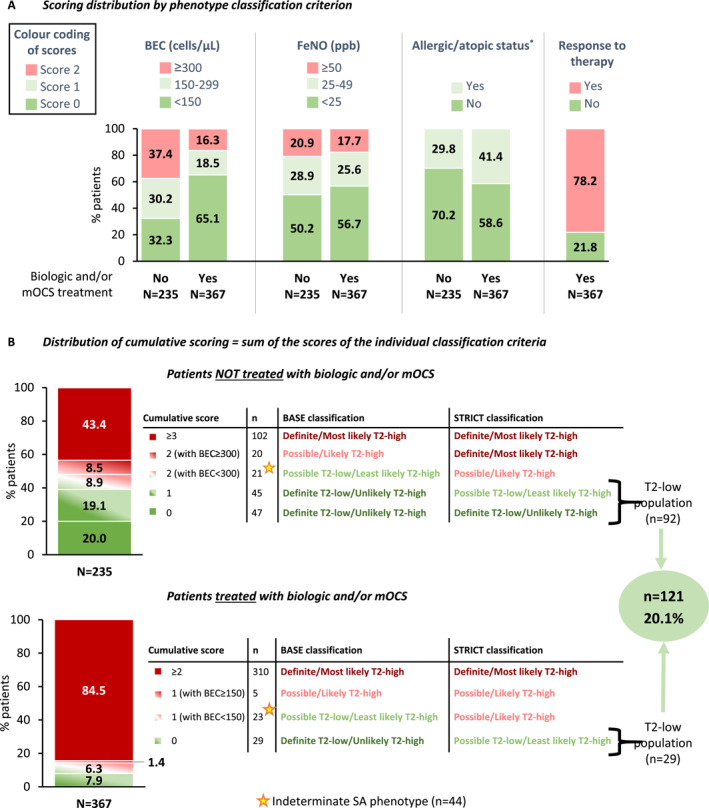
Frequency of T2‐low SA based on the composite scoring system for SA phenotype classification. Patient distribution per (A) score and (B) cumulative score, in the subpopulations per current receipt of biologic and/or OCS treatment. *Defined as fulfillment of at least 2 of the following: Total Immunoglobulin *E* ≥ 100 IU/mL; skin prick test positivity; Early‐onset asthma (<18 years old). BEC, blood eosinophil count; FeNO, fractional exhaled nitric oxide; mOCS, maintenance oral corticosteroids; *n*, number of patients with variable; *N*, number of patients with available data; SA, severe asthma; T2, type‐2.

With respect to T2‐inflammation biomarkers that comprised two of the four endotype classification criteria in the composite scoring system, BEC <300cells/μL and FeNO <50 ppb was reported in 75.4% (454/602) and 81.1% (488/602) of the patients overall, respectively. Overall, 63.1% (380/602) of patients had a positive allergic/atopic status. Since different thresholds were employed for classifying the SA endotype of patients currently receiving biologics/mOCS, the scores distribution is presented in Figure 1A separately among those treated and not treated with biologics/mOCS. Of those treated with biologics/mOCS, 21.8% (80/367) were non‐responders to therapy.

The number of patients corresponding to each unique combination of endotype classification criteria threshold along with respective cumulative score is provided in Table S1. Among patients not treated with biologics/mOCS, 48.1% (113/235) and 39.1% (92/235) had T2‐low SA based on the BASE and STRICT definitions, respectively (top panel of Figure 1B and Figure S2). Among those treated with biologics/mOCS, 14.2% (52/367) and 7.9% (29/367) had T2‐low SA based on the BASE and STRICT definitions, respectively (bottom panel of Figure 1B and Figure S2).

Discordance between the BASE and STRICT definitions was identified for 7.3% of patients, that is, *n* = 44 (21 among those not treated and 23 among those treated with biologics/mOCS), who were classified as “possible T2‐low” based on the former and “possible T2‐high” based on the latter definition (Figure 1B; Figure S2). After careful examination of the clinical profile of these intermediate endotype cases, we observed an increased frequency of T2‐high features among them, suggesting that they may represent the T2‐high rather than the T2‐low endotype; specifically, the highly prevalent T2‐high features included allergic rhinitis (63.6%), nasal polyposis (38.6%), skin prick test positivity (72.5%), and current treatment with biologics (47.7%). Since these cases were misclassified as T2‐low using the BASE definition, the foregoing advocates that the actual T2‐low endotype is more reliably detected by the STRICT classification scheme. After excluding the 44 (7.3%) intermediate SA endotype cases, the frequency of T2‐low SA was 20.1% (121/602; 95% CI: 16.97–23.53), whereas the rate of T2‐high SA was 72.6% (437/602).

**FIGURE 2:**
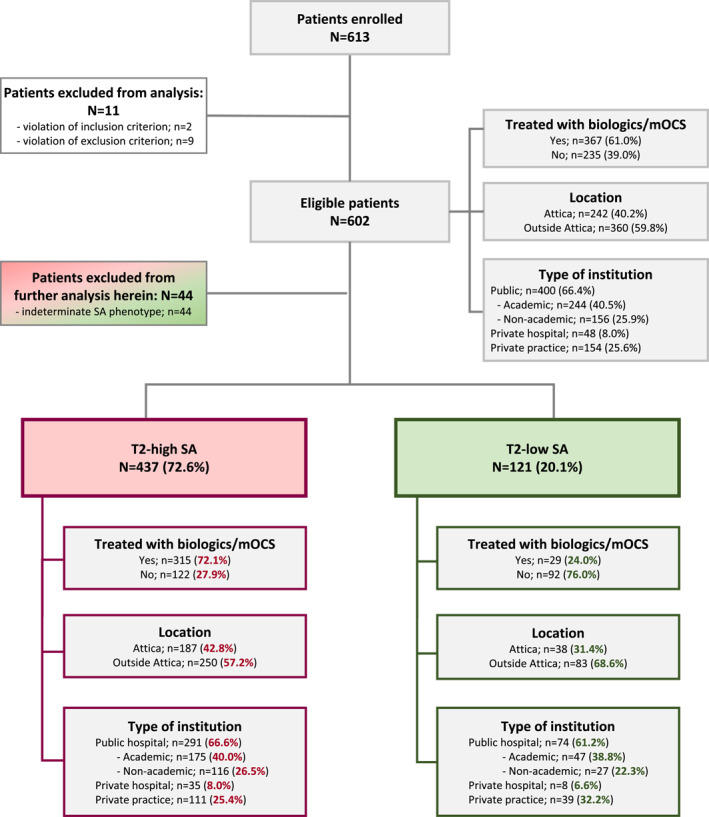
Eligible patient disposition by SA phenotype, treatment with biologics and/or mOCS, type of healthcare institution and site location. mOCS, maintenance oral corticosteroids; *n*, number of patients with variable; *N*, number of patients with available data; SA, severe asthma; T2, type‐2.

Patient disposition in terms of biologics/mOCS treatment, healthcare institution type and location are depicted in Figure 2.

### 3.3 Characteristics and exacerbation burden in the T2‐low and T2‐high subpopulations

Patient characteristics are presented in Table 1 and Table S2 for the T2‐low and T2‐high subpopulations, respectively. Median (interquartile range; IQR) asthma duration at the study visit was 10.9 (4.5–20.2) and 12.0 (5.4–22.9) years in the T2‐low and T2‐high subpopulations, respectively. The most recent biomarker (BEC, FeNO, IgE) levels in the two subpopulations are provided in Figure S3. As per study design, further sociodemographic/clinical characteristics, including lung function parameters, were collected for the T2‐low subpopulation (study part B) (Table 1).

**TABLE 1:** Sociodemographic and clinical characteristics of the overall T2‐low SA population and its subpopulations by ACT‐based asthma control level.

Characteristic^a^	Overall *N* = 121	Controlled *N* = 45	Uncontrolled *N* = 76
Sociodemographic characteristics			
Age at study visit, mean (SD)	57.2 (15.8)	56.6 (16.1)	57.5 (15.7)
Females, % (*n*/*N*)	71.1 (86/121)	73.3 (33/45)	69.7 (53/76)
Urban residence, % (*n*/*N*)	57.9 (70/121)	66.7 (30/45)	52.6 (40/76)
Education ≥13 years, % (*n*/*N*)	47.9 (58/121)	55.6 (25/45)	43.4 (33/76)
Employed, % (*n*/*N*)	46.3 (56/121)	42.2 (19/45)	48.7 (37/76)
BMI, median (IQR)	27.1 (24.0–32.9)	27.2 (23.9–34.0)	26.6 (24.0–32.6)
Former smokers, % (*n*/*N*)	29.8 (36/121)	26.7 (12/45)	31.6 (24/76)
with ≥10 pack‐years	17.4 (21/121)	17.8 (8/45)	17.1 (13/76)
Alcohol use >2 units/week, % (*n*/*N*)	10.7 (13/121)	15.6 (7/45)	7.9 (6/76)
Physically inactive, % (*n*/*N*)	58.7 (71/121)	55.6 (25/45)	60.5 (46/76)
Clinical characteristics			
Adult‐onset asthma, % (*n*/*N*)	88.4 (107/121)	91.1 (41/45)	86.8 (41/76)
Age at initial asthma diagnosis, mean (SD)	42.9 (16.9)	45.8 (17.5)	41.2 (16.4)
Age at SA diagnosis, mean (SD)	52.6 (15.5)	52.4 (15.8)	52.7 (15.4)
Time from SA diagnosis to visit, median (IQR), years	3.1 (1.2–5.0)	3.1 (1.1–4.9)	3.0 (1.7–5.1)
Family history of asthma/atopy, % (*n*/*N*)	24.0 (29/121)	17.8 (8/45)	27.6 (21/76)
History of positive SPT, % (*n*/*N*)	22.4 (19/85)	24.1 (7/29)	21.4 (12/56)
Rescue systemic steroid use last year, % (*n*/*N*)	39.7 (48/121)	17.8 (8/45)	52.6 (40/76)
Uncontrolled asthma per ATS/ERS, % (*n*/*N*)	72.7 (88/121)	26.7 (12/45)	100.0 (76/76)
Non‐asthma related active comorbidities, % (*n*/*N*)	24.8 (30/121)	33.3 (15/45)	19.7 (15/76)
Comorbidities and/or risk factors contributing to asthma symptoms and exacerbations (reported in >3 patients)^b^			
≥1 comorbidity and/or risk factor, % (*n*/*N*)	76.9 (93/121)	80.0 (36/45)	75.0 (57/76)
Allergic rhinitis	41.3 (50/121)	46.7 (21/45)	38.2 (29/76)
Chronic rhinosinusitis	31.4 (38/121)	35.6 (16/45)	28.9 (22/76)
Gastroesophageal reflux disease	28.9 (35/121)	26.7 (12/45)	30.3 (23/76)
Environmental tobacco exposure	21.5 (26/121)	22.2 (10/45)	21.1 (10/76)
Cardiac disease	14.0 (17/121)	17.8 (8/45)	11.8 (9/76)
Bronchiectasis	13.2 (16/121)	13.3 (6/45)	13.2 (10/76)
Eczema	10.7 (13/121)	11.1 (5/45)	10.5 (8/76)
History of food allergy or anaphylaxis	9.9 (12/121)	13.3 (6/45)	7.9 (6/76)
Nasal polyposis	9.9 (12/121)	17.8 (8/45)	5.3 (4/76)
Obstructive sleep apnea	9.9 (12/121)	8.9 (4/45)	10.5 (8/76)
History of occupational asthma	7.4 (9/121)	6.7 (3/45)	7.9 (6/76)
Prior nasal surgery	7.4 (9/121)	8.9 (4/45)	6.6 (5/76)
Other environmental exposures at home/work including allergens	6.6 (8/121)	4.4 (2/45)	7.9 (6/76)
Kyphosis due to osteoporosis	5.0 (6/121)	.	7.9 (6/76)
Pre‐bronchodilator spirometric measurements within 3 months before or at the study visit			
FEV_1_ (% predicted), median (IQR)	78.0 (65.0–94.0)	84.0 (73.0–98.0)	72.5 (56.5–92.5)
FEV_1_<65% predicted (PB), % (*n*/*N*)	24.8 (30/121)	8.9 (4/45)	34.2 (26/76)
FEV_1_ (absolute, L), median (IQR)	2.1 (1.6–2.8)	2.2 (1.8–3.1)	2.1 (1.5–2.7)
FVC (percent predicted, %), mean (SD)	86.0 (19.6)	91.1 (16.3)	82.9 (20.8)
FVC (absolute, L), median (IQR)	2.8 (2.2–3.7)	2.8 (2.2–3.9)	2.9 (2.1–3.5)
FEV_1_/FVC ratio (PB), median (IQR)	0.8 (0.6–0.8)	0.8 (0.7–0.8)	0.8 (0.6–0.8)
FEF_25‐75_ (%), median (IQR)	61.0 (40.0–75.0)	61.0 (49.0–77.0)	61.0 (34.0–75.0)
Allergy/atopy testing performed during the past 12 months			
≥1 allergy/atopy testing, % (*n*/*N*)	24.8 (30/121)	13.3 (6/45)	31.6 (24/76)
Positive for any inhaled allergen among tested patients, % (*n*/*N*)	31.0 (9/29)	66.7 (4/6)	21.7 (5/23)

*Note*: The normality of distribution of continuous variables was examined using the Shapiro‐Wilk test.

Abbreviations: ACT, asthma control test; ATS/ERS, American thoracic society/European respiratory society; BMI, body mass index; FEF, forced expiratory flow; FEV_1_, forced‐expiratory volume in 1 second; FVC, forced vital capacity; IQR, interquartile range; *N*, number of patients with available data; *n*, number of patients with variable; PB, pre‐bronchodilator; SA, severe asthma; SD, standard deviation; SPT, skin prick test; T2, type‐2.

^a^ For variables not following a normal distribution in at least one of the study subpopulations, a uniform presentation of median (IQR) was applied.

^b^ Chronic Obstructive Pulmonary Disease, *n* = 3; obesity, *n* = 2; anxiety disorder, atopic dermatitis, history of perimenstrual (catamenial) asthma, hypothyroidism, infections, Parkinson's disease, urticaria, *n* = 1, each.

**FIGURE 3:**
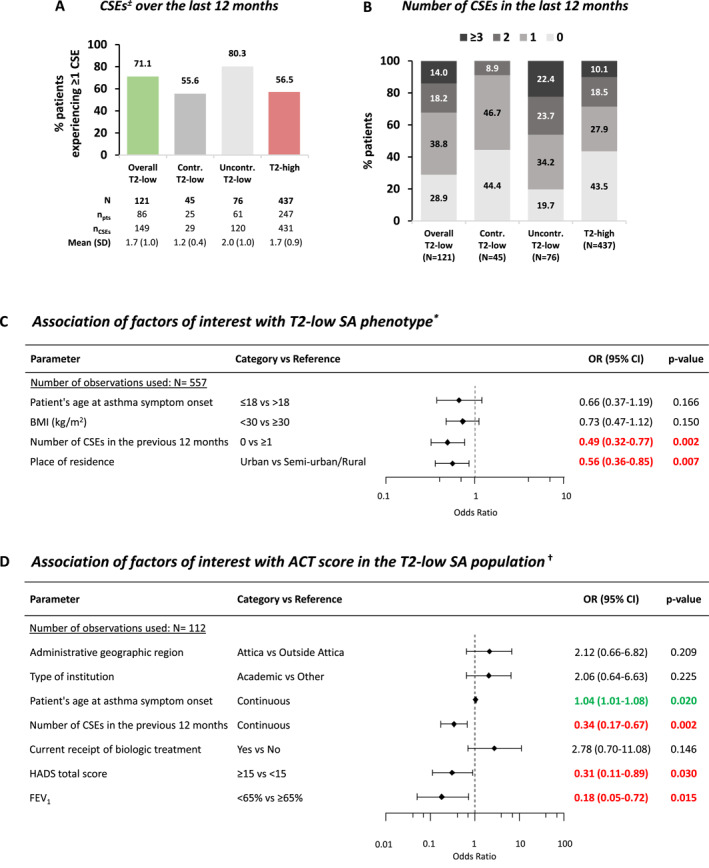
CSEs in the overall T2‐low SA population, its subpopulations by ACT‐based asthma control level, and the T2‐high SA population (A, B). Association through multivariable logistic regression models of factors of interest with (C) T2‐low SA phenotype, and (D) ACT score in the T2‐low SA population. *CSEs are defined as a worsening of asthma symptoms that required any of the following: (a) treatment with systemic corticosteroids for at least 3 days; (b) an increase of the maintenance dose of oral corticosteroids for at least 3 days or a single depo‐injectable dose of corticosteroids; (c) an emergency department visit that required use of systemic corticosteroids; (d) hospitalization. †The modeled probability was T2‐low SA phenotype: “Yes” versus “No”. The following variables were entered in the initial step of the stepwise procedure: Patient's age at study visit (≤65 vs. > 65), Patient's age at asthma symptom onset (≤18 vs. > 18), Patient's age at SA diagnosis (continuous), BMI (kg/m^2^) (<30 vs. ≥ 30), Number of CSEs in the previous 12 months (0 vs. ≥ 1), Place of residence, Sex, Smoking status (Former smokers with ≥10 pack‐years vs. Other). The type of institution and administrative region were *not* identified as confounders based on multivariable analyses (data not shown). Hosmer‐Lemeshow *p*‐value = 0.2373 (at *α* = 0.05 we fail to reject the hypothesis that the data fit the model, therefore we conclude that the model provides adequate fit). ^‡^The modeled probability was ‘ACT ≥ 20 Controlled’ versus ‘ACT < 20 Uncontrolled’. The following variables were entered in the initial step of the stepwise procedure: Patient's age at study visit (continuous), Patient's age at asthma symptoms (continuous), BMI (kg/m2) (continuous), Number of CSEs in the previous 12 months (continuous), Place of residence, Sex, Nasal polyposis, Allergic rhinitis, Adherence to asthma treatment and correct inhaler technique, Current receipt of biologic treatment, HADS total score (≥15 vs. < 15), FEV1, BEC (cells/μL) (continuous), IgE (IU/mL) (<150 vs. other). The following covariates were not included in the analysis due to quasi/complete separation issues and/or extremely unbalanced groups: Number of CSEs in the previous 12 months (≤2 vs. > 2 or ≤3 vs. > 3) and Current receipt of mOCS treatment (Yes vs. No). Type of institution and administrative region were identified as *confounders* based on multivariable analyses (data not shown), thus were excluded from the stepwise process, and forced in the final model. Hosmer‐Lemeshow *p*‐value = 0.99 (at *α* = 0.05 we fail to reject the hypothesis that the data fit the model, therefore we conclude that the model provides adequate fit). ACT, asthma control test; BMI, body mass index; CI, confidence interval; CSE, clinically significant exacerbation; FEV_1_, forced‐expiratory volume in 1 s; HADS, hospital anxiety and depression scale; *n*_CSEs_, number of clinically significant exacerbations; *n*_pts_, number of patients; *N*, number of patients with available data; OR, odds ratio; SA, severe asthma; SD, standard deviation; T2, type‐2.

Over the 12 months before enrollment, 71.1% and 56.5% of the T2‐low and T2‐high SA subpopulations, respectively, had experienced ≥1 clinically significant exacerbation (CSE) (Figure 3A), with 32.2% and 28.6% having experienced ≥2 CSEs (Figure 3B). The respective 12‐month CSE rates were 1.23 (95% CI: 1.05–1.45) and 0.99 (95% CI: 0.90–1.08) episodes/patient‐year.

The association of factors of interest with frequency of T2‐low SA endotype was examined using univariable (Table S3) and multivariable (Figure 3C) regression analyses. Based on multivariable analysis, patients without any CSEs in the previous year and patients residing in urban areas had lower odds of having T2‐low SA endotype than those with ≥1 CSE and those residing in semi‐urban/rural areas (Odds Ratio: 0.49 and 0.56, respectively; Figure 3C).

### 3.4 Asthma symptom control in the T2‐low subpopulation

In the T2‐low SA subpopulation, median (IQR) ACT score at the study visit was 18.0 (16.0–21.5), with 37.2% (45/121; 95% CI: 28.58–46.44) of patients having well‐controlled (ACT score: 20–25), 39.7% (48/121; 95% CI: 30.89–48.96) not well‐controlled (ACT: 16–19), and 23.1% (28/121; 95% CI: 15.96–31.68) very poorly controlled asthma (ACT: 5–15); the latter two comprised the “uncontrolled” subgroup (62.8%).

The characteristics of the subgroups of patients by ACT‐defined asthma control level are presented in Table 1 and Figure S3. CSEs over the past 12 months before the study visit in the “controlled” and “uncontrolled” subgroups are shown in Figure 3; CSE rates were 0.64 (95% CI: 0.45–0.93) and 1.58 (95% CI: 1.32–1.89) episodes/patient‐year, respectively.

The association of factors of interest with asthma symptom control among T2‐low SA patients was examined using univariable (Table S4) and multivariable (Figure 3D) regression analyses. Multivariable analysis determined the following factors as predictors of uncontrolled asthma (ACT<20): increasing number of CSEs in the previous year, HADS‐total (HADS‐T) score ≥15 and forced‐expiratory volume in 1 s (FEV_1_) <65% predicted. On the contrary, increasing patient's age at asthma symptom onset was positively associated with well‐controlled asthma (Figure 3D).

### 3.5 Current asthma medication in the T2‐low and T2‐high subpopulations

**FIGURE 4:**
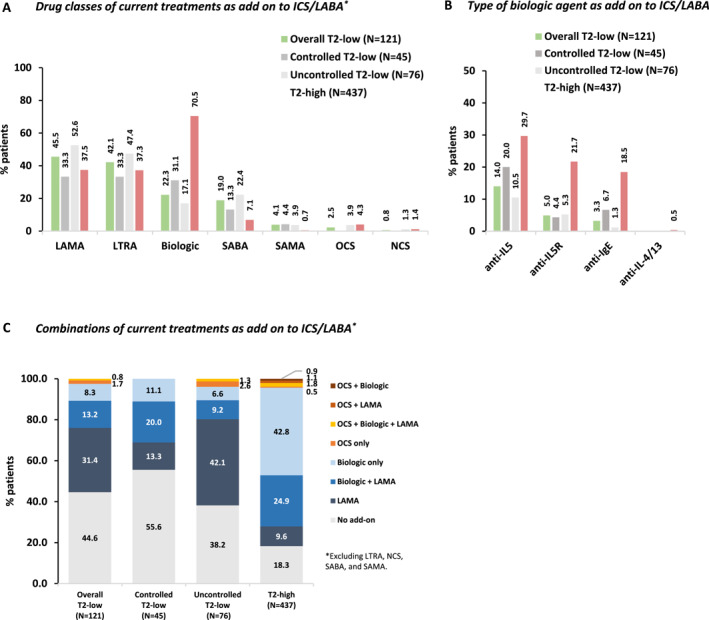
Current management strategies in the overall T2‐low SA population, its subpopulations by ACT‐based asthma control level, and the T2‐high SA population. *Macrolide antibiotic was used in 1 (0.2%) T2‐high patient. †Excluding LTRA, NCS, SABA, and SAMA. ACT, asthma control test; ICS, inhaled corticosteroids; IgE, immunoglobulin E; IL, interleukin; IL‐5R, interleukin‐5 receptor; LABA, long‐acting beta agonist; LAMA, long‐acting muscarinic antagonist; LTRA, leukotriene receptor antagonist; *N*, number of patients with available data; NCS, nasal corticosteroids; OCS, oral corticosteroids; SA, severe asthma; SABA, short‐acting β‐agonist; SAMA, short‐acting muscarinic antagonist; T2, type‐2.

**FIGURE 5:**
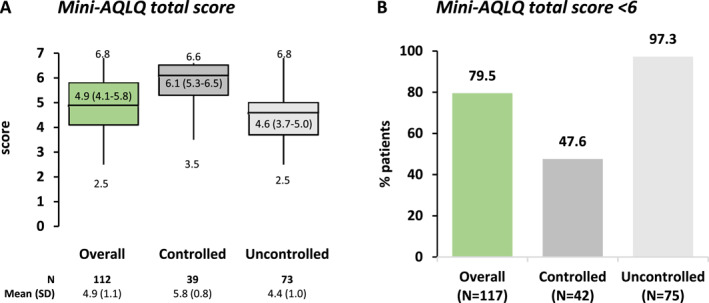
Asthma‐specific QoL at the study visit, in the overall T2‐low SA and its subpopulations by ACT‐based asthma control level. Box‐plots depict median with IQR, including whiskers that extend from minimum to maximum values. ACT, asthma control test; AQLQ, asthma quality of life questionnaire; IQR, interquartile range; *N*, number of patients with available data; QoL, quality of life; SA, severe asthma; SD, standard deviation; T2, type‐2.

Current receipt of any add‐on medication (as maintenance and/or reliever) to high‐dose ICS/long‐acting beta agonist (LABA) was reported for 80.2% (97/121) and 92.0% (402/437) of the T2‐low and T2‐high SA patients, respectively. The most frequent add‐on treatments were long‐acting muscarinic antagonists (LAMA), leukotriene receptor antagonists (LTRA) and/or biologic agents (Figure 4A), with anti‐IL5(R) agents accounting for most biologics (Figure 4B). Treatment patterns are depicted in Figure 4C. Biologic add‐on treatment was given to 22.3% of T2‐low patients [median (IQR) exposure: 1.8 (1.0–4.2) years] and was more common among T2‐low controlled patients; as expected, this frequency was much higher among T2‐high SA patients [70.5%; median (IQR) exposure: 1.8 (0.9–3.8) years] (Figures 4A and 5C). Only 1/121 T2‐low SA patients (0.8%) and 13/437 T2‐high patients (3.0%) were OCS‐dependent (i.e., treated with OCS equivalent to a daily dose of ≥5 mg prednisone for ≥3 months). Allergen immunotherapy was reported in 6.2% of the T2‐high SA patients.

Physician‐reported patients' adherence to both maintenance medication and correct inhaler technique was “high” for 78.5% of the T2‐low and 84.0% of the T2‐high SA patients.

### 3.6 PROs and HCRU in the T2‐low subpopulation

HRQoL of T2‐low SA patients was impaired with median total Mini‐AQLQ score of 4.9 (Figure 5A), the most affected domain being “environmental stimuli” (Figure S4A), and a score <6 reported in 79.5% (93/117) (Figure 5B). Median (IQR) anxiety (HADS‐A) and depression (HADS‐D) subscale scores were 7.0 (4.0–10.0) and 6.0 (2.0–9.0), respectively. The median HADS‐T score was 13.0, with 43.8% (49/112) of patients having clinically significant emotional distress (score ≥15) (Figure S4B‐C).

All but one (120/121) T2‐low SA patient completed the WPAI:RS questionnaire at the study visit and reported a median AI of 30.0% (Figure S5). Employed patients reported a median (IQR) absenteeism, presenteeism and work productivity loss (WPL) of 0.0 (0.0–4.8)%, 30.0 (0.0–50.0)%, and 30.0 (5.0–51.4)%, respectively (Figure S5A).

HCRU outcomes are presented in Figure S5B. The rate of asthma‐related ED visits over the last year was 0.21 (95% CI: 0.14–0.31) for the overall T2‐low SA population, and 0.07 (95% CI: 0.02–0.21) and 0.29 (95% CI: 0.19–0.44) for the controlled and uncontrolled subgroups, respectively. The annual asthma‐related hospitalization rate was 0.04 (95% CI: 0.02–0.10) for the overall T2‐low SA population (all had uncontrolled asthma), with the median total length of hospital stay being 7.0 (range: 5.0–8.0) days.

Relevant information on PROs and HCRU outcomes among controlled and uncontrolled subgroups is provided in Figure 5, Figures S4 and S5, indicating that patients with uncontrolled T2‐low SA have worse PROs and more asthma‐related health care encounters.

## 4 DISCUSSION

The PHOLLOW study proposes a novel tool for classification of SA patients in T2‐low and T2‐high endotypes and provides valuable insights into the frequency, characteristics, treatment landscape and humanistic burden of the T2‐low SA endotype in Greece.

Our scoring tool is based on the biomarkers blood eosinophils and FeNO and allergic/atopic status. For patients currently receiving biologic and/or mOCS treatment, the scoring system employed lower thresholds for the biomarkers due to the effect of treatment on them, but we also took into consideration the response to such treatment. We elaborated two scores—BASE and STRICT—using the later lower cut‐off levels for the biomarkers. Our scoring system has not been validated elsewhere and accordingly when we evaluated our findings using both definitions—BASE and STRICT—in parallel with the clinical characteristics of the patients, we realized that the STRICT definition represents more accurately the T2‐low population by not including possible T2‐high asthmatics that would have been categorized as T2‐low with the BASE definition. The biomarker cut‐offs included in the scoring tool for defining T2 high/T2 low asthma were clear and were reviewed in each asthmatic patient separately, irrespective of the specialty (pulmonologist or allergist) that evaluated the patient.

Based on a composite measure of airway inflammation biomarkers (BECs and FeNO), which also took into consideration patients' allergic/atopic status, and response to biologic/mOCS treatment, we found that a fifth of SA patients are classified into the T2‐low endotype. This rate resembles the majority of respective rates reported in the literature for SA, despite variations in classification schemes: 34% (Sweden; T2‐low based on: BEC < 300 cells/μL & FeNO < 25 ppb & IgE < 150 IU/mL),^25^ 24% (UK; BEC ≤ 150 cells/μL & FeNO ≤ 20 ppb),^24^ 23% (UK; BEC < 150 cells/μL & FeNO < 15 ppb & periostin < 45 ng/ML),^23^ 20% (UK; BEC < 300 cells/μL & FeNO < 25 ppb),^22^ 18% (Finland; uncontrolled SA; BEC < 150 cells/μL & FeNO < 25 ppb),^21^ 18% (Japan; BEC < 150 cells/μL & IgE < 75 IU/mL),^20^ 12% [International SA Registry (ISAR); BEC < 300 cells/μL & FeNO < 25 ppb & IgE < 75 kU/L],^19^11% (Japan; uncontrolled SA; absence of atopy & BEC ≤ 300 cells/μL & FeNO ≤ 25 ppb)^18^ and 9% (UK; BEC < 150 cells/μL & FeNO < 25 ppb).^17^ Our findings expand on the limited available European literature and yield evidence of a multicomponent tool for T2‐high and T2‐low endotype‐driven classification. Our tool can be useful in the establishment of a much needed universal and precise definition of the T2‐low endotype, which is currently lacking but is critical toward personalized care delivery in SA. Furthermore, in view of the expanding therapeutic armamentarium, the present preliminary characterization of the real‐world T2‐low subpopulation can inform healthcare decision makers about the generalizability and relevance of clinical trial data.

One of the identified predictors of T2‐low endotype was non‐urban residence, which can be speculatively linked to T2‐high markers in urban areas due to increased perennial allergen sensitization caused by air pollution.^40^ Furthermore, patients experiencing ≥1 CSEs in the past year had higher odds of having T2‐low SA. Notably, mean number of CSEs over the previous year was 1.7 in the PHOLLOW T2‐low SA subpopulation, which is the same as reported previously in the ISAR regardless of endotype.^41^ In the latter study, the use of biologics, which have demonstrated efficacy in reducing exacerbations in eosinophilic asthma,^42^, ^43^ was low (<26%). In addition, the CSE rate of 1.23 episodes/year in PHOLLOW T2‐low SA patients falls within the range of 1.22–1.65 reported for the subgroups of patients in the placebo arm of tezepelumab trials fulfilling ≥2 T2‐low biomarker thresholds (including BEC/FeNO/perennial allergy).^34^ Hence, the unsurprisingly high rate of biologic use (71%) and controlled asthma (73%) among T2‐high patients in PHOLLOW, coupled with the absence of tailored treatments for T2‐low SA, possibly explain the positive association of ≥1 CSEs with T2‐low endotype.

Symptoms remained uncontrolled (ACT score<20) in a high proportion of T2‐low SA patients in PHOLLOW (63%). These results are in line with findings from patients with general SA (any endotype) in two large international real‐world studies (65%–77%.^41^, ^44^) As in PHOLLOW, less than a third of patients were biologic‐treated in these older studies. On the other hand, in a more recent study from Swiss SA Registry in which 82% of SA patients were biologic‐treated, ACT‐defined uncontrolled asthma was reported in only 26%, while their multivariable analysis indicated a positive association between asthma control and biologics.^45^ Accordingly, in PHOLLOW, physician‐assessed uncontrolled asthma was much lower among T2‐high SA patients (28% vs. 73% in T2‐low), 71% of whom were biologic‐treated. A growing body of real‐world evidence suggests that biologics lead to reduced exacerbation frequency and improved lung function and ACT scores in T2‐high asthma.^42^, ^43^ Consistently, frequent exacerbations and worse lung function were associated with uncontrolled SA based on our multivariable analysis, corroborating the findings of previous asthma studies.^46^ Therefore, future asthma control rates are expected to improve with increasing use of existing biologics among T2‐high SA and the introduction of newer much anticipated treatments for T2‐low SA.

Of note, 22.3% of T2‐low patients in PHOLLOW were receiving biologics not licensed for T2‐low asthma, indicating the unmet therapeutic need in this subgroup and possibly an off‐label use of biologics in clinical routine.

The PHOLLOW Mini‐AQLQ score results (mean = 4.9; score <6 = 80%) suggest that HRQoL is impaired in T2‐low SA patients as in general asthmatics (any severity/any endotype) in other European countries (mean = 4.5–5.4^47^, ^48^, ^49^; score <6 = 58%^48^), general patients with asthma in other Greek cohorts (mean = 4.4–4.6^50^, ^51^), as well as severe/difficult‐to‐treat asthma patients based on data from the US TENOR and Swedish BREATHE cohorts (mean = 4.9–5.3.^25^, ^52^) Many real‐world studies have already established asthma control as a major determinant of disease‐specific QoL.^47^, ^48^, ^49^, ^52^, ^53^, ^54^, ^55^, ^56^ Consistently, the uncontrolled SA T2‐low patients in PHOLLOW had lower mean Mini‐AQLQ scores (4.6) than their controlled counterparts (6.1), similar to the literature ranges of 4.3–4.5^57^, ^58^, ^59^, ^60^ and 5.8–6.1^55^, ^57^, ^60^ for general SA (any endotype), respectively. Importantly, almost all patients in the uncontrolled and nearly half of those in the controlled T2‐low SA subgroups of PHOLLOW had a score < 6. Our results indicate that T2‐low SA, especially when uncontrolled, may result in substantial HRQoL impairments.

Our study also demonstrated clinically significant emotional distress associated with uncontrolled asthma in T2‐low SA. The rate of HADS‐T score ≥15 was more than double in patients with uncontrolled (55%) compared with controlled asthma (24%). Similarly, mean HADS‐T score was higher among uncontrolled than controlled patients (15.4 vs. 9.2), signifying higher distress than uncontrolled (12.3–13.6) and controlled (7.0) patients of the European U‐BIOPRED cohort (any endotype SA), albeit different asthma control definitions.^57^ In addition, the PHOLLOW T2‐low SA population seems especially affected by psychological distress when comparing individual HADS scores with normative data; specifically, mean HADS‐D and HADS‐A scores of 3.9 and 5.1, respectively, have been reported for the control group of a Greek HADS validation study.^61^

Median WPL and AI scores in PHOLLOW T2‐low SA patients were worse than the previously reported among Greek patients with chronic lung disease (including asthma),^62^ while mean scores were at least as high as the international NOVELTY cohort (any endotype SA; WPL: 32.4 vs. 12.1 and AI: 40.9 vs. 17.3, in uncontrolled vs. controlled SA, respectively).^44^ Furthermore, our findings corroborate the relationship between asthma control and WPL that has been documented in the literature.^44^, ^46^, ^56^, ^60^, ^63^, ^64^ Lastly, the PHOLLOW findings showed that healthcare encounters were non‐negligible, with more than half and a fifth of uncontrolled T2‐low SA patients performing asthma‐related unscheduled private practice and ED visits in the past year, respectively.

PHOLLOW had some limitations inherent to its cross‐sectional/retrospective design (including potential bias with respect to patient selection, systematic error, measurement, and reporting), and the mechanisms utilized to mitigate these foreseen limitations have been described previously.^38^ Importantly, the planned sample sizes were met, lending credibility to the precision of study outcomes. Furthermore, the rate of missing data did not exceed 10% for key variables. It is noteworthy, however, that caution should be exercised when interpreting relevant observations in the absence of statistical comparisons between subgroups by asthma control level. Additionally, HCRU outcomes may be underestimated due to recall bias. Lastly, with respect to the exploratory objective of identifying T2‐low predictors, potential bias arising from residual confounding (such as environmental exposures) was not accounted for in the logistic regression analysis, as this information was only collected for the T2‐low subpopulation in the context of this study.^38^ Future analyses to strengthen and expand our findings are warranted.

In summary, we describe here for the first time a composite measure of airway inflammation, including patients' allergic/atopic status, and response to biologic/mOCS treatment, to classify SA patients in T2‐low and T2‐high endotypes. Based on two different well‐defined classification schemes, at least one‐fifth of SA patients present with a T2‐low endotype in Greece. Despite the long‐term duration of high‐dose ICS/LABA treatment and high adherence, symptoms remain uncontrolled in six out of 10 T2‐low SA patients, affecting their QoL and work ability, which, together with an increased HCRU, may contribute to substantial socioeconomic burden. In conclusion, the burden of T2‐low SA is significant, highlighting the unmet need in this patient population who would benefit from upcoming targeted therapeutic approaches. The results of the present study can serve as a benchmark for evaluating the uptake of new therapeutic approaches in clinical practice and associated outcomes.

## AUTHOR CONTRIBUTIONS

**Konstantinos Porpodis**: Resources, investigation, writing—review & editing. **Nikolaos Zias**: Investigation, resources, writing—review & editing. **Konstantinos Kostikas**: Investigation, writing—review & editing. **Argyris Tzouvelekis**: Investigation, writing—review & editing. **Michael Makris**: Investigation, writing—review & editing. **George N. Konstantinou**: Writing—review & editing, investigation. **Eleftherios Zervas**: Investigation, writing—review & editing. **Stelios Loukides**: Investigation, writing—review & editing. **Paschalis Steiropoulos**: Investigation, writing—review & editing. **Konstantinos Katsoulis**: Investigation, writing—review & editing. **Anastasios Palamidas**: Investigation, writing—review & editing. **Aikaterini Syrigou**: Investigation, writing—review & editing. **Maria Gangadi**: Investigation, writing—review & editing. **Antonios Christopoulos**: Investigation. **Dimosthenis Papapetrou**: Investigation. **Fotios Psarros**: Investigation. **Konstantinos Gourgoulianis**: Investigation. **Eleni Tzortzaki**: Investigation. **Stylianos K. Vittorakis**: Investigation. **Ioannis Paraskevopoulos**: Investigation. **Ilias Papanikolaou**: Investigation. **Georgios Krommidas**: Investigation. **Dimitrios Latsios**: Investigation. **Nikolaos Tzanakis**: Investigation. **Miltiadis Markatos**: Investigation. **Angeliki Damianaki**: Investigation. **Argyrios Manikas**: Investigation. **Alexia Chatzipetrou**: Investigation. **Dimitrios Vourdas**: Investigation. **Ioanna Tsiouprou**: Writing—review & editing, investigation. **Christina Papista**: Writing—original draft, formal analysis, writing—review & editing. **Marina Bartsakoulia**: Writing—review & editing. **Nikolas Mathioudakis**: Writing—review & editing, methodology. **Petros Galanakis**: Conceptualization, methodology. **Petros Bakakos**: Supervision, conceptualization, methodology, investigation, writing—review & editing, writing—original draft, formal analysis, funding acquisition.

## CONFLICT OF INTEREST STATEMENT

K. Porpodis has received grants or contracts from Boehringer Ingelheim, Menarini, AstraZeneca, GSK, Elpen and Pfizer; consulting fees from Boehringer Ingelheim, Menarini, AstraZeneca, GSK, Elpen and Pfizer; payment or honoraria from Boehringer Ingelheim, Menarini, AstraZeneca, GSK, Elpen and Pfizer; payment for expert testimony from Boehringer Ingelheim, Menarini, AstraZeneca, GSK, Elpen and Pfizer; and support for attending meetings and/or travel from Boehringer Ingelheim, Menarini, AstraZeneca, GSK, Elpen and Pfizer. K. Kostikas has received grants or contracts from AstraZeneca, Boehringer Ingelheim, Chiesi, Innovis, Elpen, GSK, Menarini, Novartis and NuvoAir; has received consulting fees from AstraZeneca, Boehringer Ingelheim, Chiesi, Csl Behring, Elpen, GSK, Menarini, Novartis, Pfizer and Sanofi Genzyme; has received payment or honoraria from Alector Pharmaceuticals, AstraZeneca, Boehringer Ingelheim, Chiesi, Elpen, Gilead, GSK, Menarini, MSD, Novartis, Sanofi Genzyme, Pfizer and WebMD; has participated in Data Safety Monitoring Board or Advisory Board for Chiesi; and is a member of GOLD Assembly. A. Tzouvelekis has received grants or honoraria from Boehringer Ingelheim, Hoffman La Roche, Menarini, AstraZeneca, GSK, Elpen, Pfizer and Bayer (financial support for department); is holder of therapeutic patents “Inhaled or aerosolized delivery of thyroid hormone to the lung as a novel therapeutic agent in fibrotic lung diseases, OCR#6368“ and “Administering Thyroid receptor b‐agonist hormone for preventing or treating a fibrotic lung disease, OCR#20230372275“ both disclosed to Yale University (both outside the submitted work); has received consulting fees from Boehringer Ingelheim, Hoffman La Roche, Menarini, AstraZeneca, GSK, Elpen, Pfizer, Pliant, Puretech, Guidotti and Genentech (outside the submitted work); has received payment or honoraria from Boehringer Ingelheim, Hoffman La Roche, Menarini, AstraZeneca, GSK, Elpen, Pfizer, Bayer, Sobi and Gilead (outside the submitted work); has received payment for expert testimony for Boehringer Ingelheim, Hoffman La Roche, Elpen, Pliant and Puretech (outside the submitted work); and has received support for attending meetings and/or travel from Boehringer Ingelheim, Hoffman La Roche, Menarini, AstraZeneca, GSK, Elpen and Pfizer. M. Makris has received grants or contracts from GSK, Chiesi, Sanofi Aventis, AstraZeneca, Elpen, Pfizer and Abbvie; consulting fees from GSK, Chiesi, Sanofi Aventis, AstraZeneca, Elpen and Pfizer (personal); payment or honoraria from GSK, Chiesi, Sanofi Aventis, AstraZeneca, Elpen, Pfizer, Abbvie and Takeda (personal); and support to attend meetings and/or travel from Chiesi (personal and staff members), Menarini (staff members) and Takeda (personal). G. Konstantinou has received payment or honoraria from AstraZeneca, Chiesi, Menarini, Sanofi, Novartis and Vianex. E. Zervas has received advisory board fees from AstraZeneca, Chiesi, Elpen, GSK, Menarini, MSD and Novartis; has received honoraria and fees for lectures from AstraZeneca, Boehringer Ingelheim, Bristol Myers, Chiesi, Elpen, GSK, Menarini, MSD and Novartis; has received travel accommodations and meeting expenses from AstraZeneca, Chiesi, GSK and Roche; and is treasurer of the Hellenic Thoracic Society. S. Loukides has received a grant from AstraZeneca (support for department); has received payment or honoraria from AstraZeneca, GSK, Menarini, Chiesi and Elpen and has participated in an advisory board for AstraZeneca, GSK, Chiesi and Menarini. P. Steiropoulos has received consulting fees from AstraZeneca, Boehringer Ingelheim, Chiesi, Elpen, GSK, Guidotti and Menarini; payment or honoraria from AstraZeneca, Boehringer Ingelheim, Chiesi, Elpen, GSK, Guidotti, Menarini and Pfizer; and support for attending meetings and/or travel from AstraZeneca, Boehringer Ingelheim, Chiesi, Elpen, GSK, Guidotti, Menarini and Pfizer. K. Katsoulis has received payment or honoraria from GSK and support for attending meetings and/or travel from Menarini Hellas. E. Syrigou has received payment or honoraria from AstraZeneca and Menarini. M. Gangadi has received support to attend meetings and/or travel from AstraZeneca and Chiesi. A. Christopoulos has received honoraria for lectures and educational events from GSK, AstraZeneca, Menarini and Chiesi, and support for attending scientific meetings from GSK and Menarini. D. Papapetrou has received consulting fees from AstraZeneca, Menarini, Roche and GSK; payment or honoraria from AstraZeneca and Menarini and support for attending meetings and/or travel from Menarini, Elpen, Chiesi, Guidotti, AstraZeneca, Novartis, Lilly, Boehringer Ingelheim, Faran and Bayer. E. Tzortzaki has received grants or contracts from AstraZeneca, Chiesi, GSk, Elpen and Menarini (private practice), payment or honoraria from AstraZeneca, Chiesi, GSK, Elpen and Menarini (private practice); and support for attending meetings and/or travel from AstraZeneca and GSK. I. Papanikolaou has received grants or contracts from Boehringer Ingelheim, Elpen, GSK, AstraZeneca and Menarini (for institution), payment or honoraria from Boehringer Ingelheim, GSK and AstraZeneca, and support for attending meetings and/or travel from Chiesi, Boehringer Ingelheim and AstraZeneca. G. Kromidas has received support from AstraZeneca (personal). D. Latsios has received consulting fees from Chiesi, GSK, Menarini, payment or honoraria from Chiesi, Elpen, GSK, Menarini and AstraZeneca, and support for attending meetings and/or travel from Menarini and Boehringer Ingelheim. N. Tzanakis has received consulting fees from AstraZeneca, Chiesi, GSK, Guidotti, Menarini, Boehringer Ingelheim, Special Therapeutics, Pfizer and Gilead; payment or honoraria from AstraZeneca, Chiesi, GSK, Guidotti, Menarini, Boehringer Ingelheim, Special Therapeutics and Pfizer; and support for attending meetings and/or travel from AstraZeneca, Chiesi, GSK, Guidotti, Menarini, Boehringer Ingelheim, Special Therapeutics and Pfizer. M. Markatos has received consulting fees from AstraZeneca, Chiesi, GSk, Guidotti and Menarini, payment or honoraria from AstraZeneca, Chiesi, Elpen, GSK Guidotti and Menarini and support for attending meetings and/or travel from AstraZeneca, Chiesi, Elpen, GSK, Guidotti and Menarini. A. Damianaki has received a FeNO counter from AstraZeneca; grants or contracts from Chiesi; support to attend meetings and/or travel from Chiesi, Guidotti, AstraZeneca, Elpen, Menarini and Viatris; and sleep recorders for sleep lab from Menarini. A. Chatzipetrou has received payment or honoraria from GSK, Pfizer, Sanofi Aventis and AstraZeneca and support for attending meetings and/or travel from Sanofi, Vianex and Pfizer. C. Papista, M. Bartsakoulia, N. Mathioudakis, and P. Galanakis are employees of AstraZeneca Greece. P. Bakakos has received consulting fees from GSK, Menarini, Elpen, AstraZeneca, Pfizer, Vianex and MSD and payment or honoraria from AstraZeneca, Chiesi, Elpen, Menarini, Gilead, GSK and Pfizer. The rest of the authors declare that they have no relevant conflicts of interest.

## Supporting information

Supporting Information S1

## Data Availability

Data sharing is not applicable to this article as no new data were created or analyzed in this study.
