# Supplementary material for: T2‐low severe asthma clinical spectrum and impact: The Greek PHOLLOW cross‐sectional study
Source: Clin Transl Allergy. 2025 Jan 29;15(2):e70035. doi: 10.1002/clt2.70035 (PMC11779522; doi:10.1002/clt2.70035)
Supplement: Supplementary file 1 — Supporting Information S1 [file CLT2-15-e70035-s001.docx]

T2-low severe asthma clinical spectrum and impact: the Greek PHOLLOW cross-sectional study

Konstantinos Porpodis^1^, Nikolaos Zias^2^, Konstantinos Kostikas^3^, Argyris Tzouvelekis^4^, Michael Makris^5^, George N Konstantinou^6^, Eleftherios Zervas^7^, Stelios Loukides^8^, Paschalis Steiropoulos^9^, Konstantinos Katsoulis^10^, Anastasios Palamidas^11^, Aikaterini Syrigou^12^, Maria Gangadi^13^, Antonios Christopoulos^14^, Dimosthenis Papapetrou^15^, Fotios Psarros^16^, Konstantinos Gourgoulianis^17^, Eleni Tzortzaki^18^, Stylianos K Vittorakis^19^, Ioannis Paraskevopoulos^20^, Ilias Papanikolaou^21^, Georgios Krommidas^22^, Dimitrios Latsios^23^, Nikolaos Tzanakis^24^, Miltiadis Markatos^25^, Angeliki Damianaki^26^, Argyrios Manikas^27^, Alexia Chatzipetrou^28^, Dimitrios Vourdas^29^, Ioanna Tsiouprou^1^, Christina Papista^30^, Marina Bartsakoulia^30^, Nikolas Mathioudakis^30^, Petros Galanakis^30^ and Petros Bakakos^31^

^1^Pulmonary Dpt., Aristotle University of Thessaloniki, G. Papanikolaou Hospital, Thessaloniki, Greece; ^2^Respiratory Dpt., Navy Hospital of Athens, Athens, Greece; ^3^Respiratory Medicine Dpt., School of Medicine, University of Ioannina, Ioannina, Greece; ^4^Dpt. of Respiratory Medicine, Medical School, University of Patras, Patras, Greece; ^5^Allergy Unit, 2^nd^ Dpt. of Dermatology and Venereology, Medical School, National and Kapodistrian University of Athens, Attikon University General Hospital, Athens, Greece; ^6^Dpt. of Allergy and Clinical Immunology, 424 General Military Training Hospital, Thessaloniki, Greece; ^7^7^th^ Respiratory Dpt., Athens Chest Hospital Sotiria, Athens, Greece; ^8^2^nd^ Respiratory Dpt., Attikon University Hospital, National and Kapodistrian University of Athens Medical School, Athens, Greece; ^9^Dpt. of Respiratory Medicine, Medical School, Democritus University of Thrace, University General Hospital, Alexandroupolis, Greece; ^10^Pulmonary Dpt., 424 Army General Hospital, Thessaloniki, Greece; ^11^Athens Medical Center-Marousi Clinic, Athens, Greece; ^12^Allergy Dpt., Sotiria General Hospital, Athens, Greece; ^13^10^th^ Dpt. of Pulmonary Medicine, Athens Chest Hospital Sotiria, Athens, Greece; ^14^Dpt. of Respiratory Medicine University Hospital Patras, Patra, Greece; ^15^Athens Medical Group, Paleo Faliro Clinic, Athens, Greece; ^16^Allergy Dpt., Athens Naval Hospital, Athens, Greece; ^17^Dpt. of Respiratory Medicine, University Hospital of Larissa, Faculty of Medicine, University of Thessaly, Larissa, Greece; ^18^Outpatient Respiratory Clinic, Heraklion, Greece; ^19^Private Practice, Chania, Greece; ^20^401 General Military Hospital Athens, Athens, Greece; ^21^Pulmonary Dpt., Corfu General Hospital, Corfu, Greece; ^22^Private Practice, Athens, Greece; ^23^Private Practice, Drama, Greece; ^24^Dpt. of Respiratory Medicine, University General Hospital of Heraklion, Laboratory of Molecular and Cellular Pneumonology, Medical School, University of Crete, Heraklion, Greece; ^25^Outpatient clinic for Pulmonary Diseases, Chania, Greece; ^26^Pulmonary and Sleep Medical Dpt., Chania General Hospital Agios Georgios, Chania, Greece; ^27^European Interbalkan Medical Center, Thessaloniki, Greece; ^28^Allergy Unit, 2^nd^ Dpt. of Dermatology and Venereology, National and Kapodistrian University of Athens, University General Hospital Attikon, Athens, Greece; ^29^Dpt. of Allergy and Clinical Immunology, 251 General Airforce Hospital, Athens, Greece; ^30^Medical Affairs Department, Respiratory and Immunology, Athens, Greece; ^31^1^st^ University Dpt. of Respiratory Medicine, National and Kapodistrian University of Athens, Athens, Greece

**Corresponding author**: Petros Bakakos, [petros44@hotmail.com](mailto:petros44@hotmail.com)

**Supporting Information**

**List of Supplementary Tables**

[**Table S1.** Patient distribution by composite scoring system. 3](#_Toc182392591)

[**Table S2.** Sociodemographic and disease characteristics in the T2-high SA population. 4](#_Toc182392592)

[**Table S3.** Association of factors of interest with T2-low SA phenotype through univariable logistic regression analysis. 5](#_Toc182392593)

[**Table S4.** Association of the ACT score with factors of interest through univariable logistic regression analysis in the T2-low population. 6](#_Toc182392594)

**List of Supplementary Figures**

[**Figure S1.** Overview of study design. 7](#_Toc182833117)

[**Figure S2.** Frequency of possible and definite T2-low and T2-high SA categories, per phenotype definition (BASE or STRICT), in the overall population and per current receipt of biologic and/or OCS treatment. 9](#_Toc182833118)

[**Figure S3.** Information on biomarkers at most recent measurement before study visit, in the overall T2-low SA population, its subpopulations by ACT-based asthma control level, and the T2-high SA population. 10](#_Toc182833119)

[**Figure S4.** (A) Asthma-specific functional impairments and (B-C) hospital anxiety and depression at the study visit, in the overall T2-low SA and its subpopulations by ACT-based asthma control level. 11](#_Toc182833120)

[**Figure S5.** (A) Work productivity and activity impairment at the study visit, and (B) asthma-related HCRU in the 12 months before study visit, in the overall T2-low SA and its subpopulations by ACT-based asthma control level. 12](#_Toc182833121)

**Table S1.** Patient distribution by composite scoring system.

| **Treatment** | **BEC (cells/μL)** | **FeNO (ppb)** | **Allergic/atopic status** | **Response to therapy*** | **Cumulative score** | **BASE Classification** | **STRICT Classification** | **N** | **T2-low** |
| --- | --- | --- | --- | --- | --- | --- | --- | --- | --- |
| **Non-biologics and non-mOCS** | **<150** | **<25** | **No** | **NA** | **0** | **Definite T2-low/Unlikely T2-high** | **Definite T2-low/Unlikely T2-high** | **47** |  |
|  | **<150** | **<25** | **Yes** | **NA** | **1** | **Definite T2-low/Unlikely T2-high** | **Possible T2-low/Least likely T2-high** | **8** |  |
|  | **<150** | **25-49** | **No** | **NA** | **1** | **Definite T2-low/Unlikely T2-high** | **Possible T2-low/Least likely T2-high** | **12** |  |
|  | **<150** | **25-49** | **Yes** | **NA** | **2** | **Possible T2-low/Least likely T2-high** | **Possible/Likely T2-high** | **3** |  |
|  | **<150** | **≥50** | **No** | **NA** | **2** | **Possible T2-low/Least likely T2-high** | **Possible/Likely T2-high** | **3** |  |
|  | **<150** | **≥50** | **Yes** | **NA** | **3** | **Definite/Most likely T2-high** | **Definite/Most likely T2-high** | **3** |  |
|  | **150-299** | **<25** | **No** | **NA** | **1** | **Definite T2-low/Unlikely T2-high** | **Possible T2-low/Least likely T2-high** | **25** |  |
|  | **150-299** | **<25** | **Yes** | **NA** | **2** | **Possible T2-low/Least likely T2-high** | **Possible/Likely T2-high** | **5** |  |
|  | **150-299** | **25-49** | **No** | **NA** | **2** | **Possible T2-low/Least likely T2-high** | **Possible/Likely T2-high** | **10** |  |
|  | **150-299** | **25-49** | **Yes** | **NA** | **3** | **Definite/Most likely T2-high** | **Definite/Most likely T2-high** | **13** |  |
|  | **150-299** | **≥50** | **No** | **NA** | **3** | **Definite/Most likely T2-high** | **Definite/Most likely T2-high** | **13** |  |
|  | **150-299** | **≥50** | **Yes** | **NA** | **4** | **Definite/Most likely T2-high** | **Definite/Most likely T2-high** | **5** |  |
|  | **≥300** | **<25** | **No** | **NA** | **2** | **Possible/Likely T2-high** | **Definite/Most likely T2-high** | **20** |  |
|  | **≥300** | **<25** | **Yes** | **NA** | **3** | **Definite/Most likely T2-high** | **Definite/Most likely T2-high** | **13** |  |
|  | **≥300** | **25-49** | **No** | **NA** | **3** | **Definite/Most likely T2-high** | **Definite/Most likely T2-high** | **19** |  |
|  | **≥300** | **25-49** | **Yes** | **NA** | **4** | **Definite/Most likely T2-high** | **Definite/Most likely T2-high** | **11** |  |
|  | **≥300** | **≥50** | **No** | **NA** | **4** | **Definite/Most likely T2-high** | **Definite/Most likely T2-high** | **16** |  |
|  | **≥300** | **≥50** | **Yes** | **NA** | **5** | **Definite/Most likely T2-high** | **Definite/Most likely T2-high** | **9** |  |
|  | | | | | | | | | |
| **Biologics and/or mOCS** | **<150** | **<25** | **No** | **Yes** | **2** | **Definite/Most likely T2-high** | **Definite/Most likely T2-high** | **59** |  |
|  | **<150** | **<25** | **Yes** | **Yes** | **3** | **Definite/Most likely T2-high** | **Definite/Most likely T2-high** | **41** |  |
|  | **<150** | **25-49** | **No** | **Yes** | **3** | **Definite/Most likely T2-high** | **Definite/Most likely T2-high** | **31** |  |
|  | **<150** | **25-49** | **Yes** | **Yes** | **4** | **Definite/Most likely T2-high** | **Definite/Most likely T2-high** | **21** |  |
|  | **<150** | **≥50** | **No** | **Yes** | **4** | **Definite/Most likely T2-high** | **Definite/Most likely T2-high** | **15** |  |
|  | **<150** | **≥50** | **Yes** | **Yes** | **5** | **Definite/Most likely T2-high** | **Definite/Most likely T2-high** | **13** |  |
|  | **150-299** | **<25** | **No** | **Yes** | **3** | **Definite/Most likely T2-high** | **Definite/Most likely T2-high** | **21** |  |
|  | **150-299** | **<25** | **Yes** | **Yes** | **4** | **Definite/Most likely T2-high** | **Definite/Most likely T2-high** | **10** |  |
|  | **150-299** | **25-49** | **No** | **Yes** | **4** | **Definite/Most likely T2-high** | **Definite/Most likely T2-high** | **6** |  |
|  | **150-299** | **25-49** | **Yes** | **Yes** | **5** | **Definite/Most likely T2-high** | **Definite/Most likely T2-high** | **7** |  |
|  | **150-299** | **≥50** | **No** | **Yes** | **5** | **Definite/Most likely T2-high** | **Definite/Most likely T2-high** | **10** |  |
|  | **150-299** | **≥50** | **Yes** | **Yes** | **6** | **Definite/Most likely T2-high** | **Definite/Most likely T2-high** | **5** |  |
|  | **≥300** | **<25** | **No** | **Yes** | **4** | **Definite/Most likely T2-high** | **Definite/Most likely T2-high** | **14** |  |
|  | **≥300** | **<25** | **Yes** | **Yes** | **5** | **Definite/Most likely T2-high** | **Definite/Most likely T2-high** | **8** |  |
|  | **≥300** | **25-49** | **No** | **Yes** | **5** | **Definite/Most likely T2-high** | **Definite/Most likely T2-high** | **6** |  |
|  | **≥300** | **25-49** | **Yes** | **Yes** | **6** | **Definite/Most likely T2-high** | **Definite/Most likely T2-high** | **9** |  |
|  | **≥300** | **≥50** | **No** | **Yes** | **6** | **Definite/Most likely T2-high** | **Definite/Most likely T2-high** | **4** |  |
|  | **≥300** | **≥50** | **Yes** | **Yes** | **7** | **Definite/Most likely T2-high** | **Definite/Most likely T2-high** | **7** |  |
|  | **<150** | **<25** | **No** | **No** | **0** | **Definite T2-low/Unlikely T2-high** | **Possible T2-low/Least likely T2-high** | **29** |  |
|  | **<150** | **<25** | **Yes** | **No** | **1** | **Possible T2-low/Least likely T2-high** | **Possible/Likely T2-high** | **16** |  |
|  | **<150** | **25-49** | **No** | **No** | **1** | **Possible T2-low/Least likely T2-high** | **Possible/Likely T2-high** | **7** |  |
|  | **<150** | **25-49** | **Yes** | **No** | **2** | **Definite/Most likely T2-high** | **Definite/Most likely T2-high** | **3** |  |
|  | **<150** | **≥50** | **No** | **No** | **2** | **Definite/Most likely T2-high** | **Definite/Most likely T2-high** | **2** |  |
|  | **<150** | **≥50** | **Yes** | **No** | **3** | **Definite/Most likely T2-high** | **Definite/Most likely T2-high** | **2** |  |
|  | **150-299** | **<25** | **No** | **No** | **1** | **Possible/Likely T2-high** | **Possible/Likely T2-high** | **5** |  |
|  | **150-299** | **<25** | **Yes** | **No** | **2** | **Definite/Most likely T2-high** | **Definite/Most likely T2-high** | **2** |  |
|  | **150-299** | **25-49** | **No** | **No** | **2** | **Definite/Most likely T2-high** | **Definite/Most likely T2-high** | **1** |  |
|  | **150-299** | **25-49** | **Yes** | **No** | **3** | **Definite/Most likely T2-high** | **Definite/Most likely T2-high** | **1** |  |
|  | **150-299** | **≥50** | **No** | **No** | **3** | **Definite/Most likely T2-high** | **Definite/Most likely T2-high** | **-** |  |
|  | **150-299** | **≥50** | **Yes** | **No** | **4** | **Definite/Most likely T2-high** | **Definite/Most likely T2-high** | **-** |  |
|  | **≥300** | **<25** | **No** | **No** | **2** | **Definite/Most likely T2-high** | **Definite/Most likely T2-high** | **2** |  |
|  | **≥300** | **<25** | **Yes** | **No** | **3** | **Definite/Most likely T2-high** | **Definite/Most likely T2-high** | **1** |  |
|  | **≥300** | **25-49** | **No** | **No** | **3** | **Definite/Most likely T2-high** | **Definite/Most likely T2-high** | **-** |  |
|  | **≥300** | **25-49** | **Yes** | **No** | **4** | **Definite/Most likely T2-high** | **Definite/Most likely T2-high** | **2** |  |
|  | **≥300** | **≥50** | **No** | **No** | **4** | **Definite/Most likely T2-high** | **Definite/Most likely T2-high** | **3** |  |
|  | **≥300** | **≥50** | **Yes** | **No** | **5** | **Definite/Most likely T2-high** | **Definite/Most likely T2-high** | **4** |  |

*Response to therapy is defined as reduction of exacerbations and/or mOCS reduction by ≥50% based on the 12-month baseline period.

Abbreviations: BEC, Blood Eosinophil Count; FeNO, Fractional exhaled Nitric Oxide; N, number of patients; mOCS, maintenance Oral Corticosteroid; T2, Type-2.

**Table S2.** Sociodemographic and disease characteristics in the T2-high SA population.

| **Characteristic** | **T2-high**  **N=437** |
| --- | --- |
| **Sociodemographic characteristics** |  |
| Age at study visit, median (IQR) | 57.0 (46.0-67.0) |
| Females, % (n/N) | 62.7 (274/437) |
| Urban residence, % (n/N) | 71.4 (312/437) |
| BMI, median (IQR) | 27.7 (24.0-31.2) |
| Former smokers, % (n/N) | 29.5 (129/437) |
| with ≥10 pack-years | 18.5 (81/437) |
| **Disease characteristics** | |
| Adult-onset asthma, % (n/N) | 82.6 (361/437) |
| Age at initial asthma diagnosis, median (IQR) | 42.0 (31.0-54.0) |
| Age at SA diagnosis, mean (SD) | 50.8 (14.6) |
| Time from SA diagnosis to visit, median (IQR), years | 4.1 (2.0-8.1) |
| Uncontrolled asthma per ATS/ERS, % (n/N) | 27.5 (120/437) |

The normality of distribution of continuous variables was examined using the Shapiro-Wilk test. The median (IQR) is presented for variables not following a normal distribution.

Abbreviations: ATS/ERS, American Thoracic Society/European Respiratory Society; BMI, Body Mass Index; IQR, Interquartile Range; N, number of patients with available data; n, number of patients with variable; SA, Severe Asthma; SD, Standard Deviation; T2, Type-2.

**Table S3.** Association of factors of interest with T2-low SA phenotype through univariable logistic regression analysis.

| **Parameter** | **Category vs Reference** | **n_pt_**  **Overall** | **n_pt_**  **T2-low** | **OR (95% CI)** | **p-value** |
| --- | --- | --- | --- | --- | --- |
| Patient's age at the study visit | Continuous | 558 | 121 | 1.00 (0.99-1.02) | 0.726 |
|  | ≤65 vs >65 | 398 vs 160 | 85 vs 36 | 0.94 (0.60-1.45) | 0.767 |
| Patient's age at asthma symptom onset | Continuous | 558 | 121 | 1.01 (0.99-1.02) | 0.301 |
|  | ≤18 vs >18 | 98 vs 460 | 16 vs 105 | 0.66 (0.37-1.18) | 0.159 |
| Patient's age at SA diagnosis | Continuous | 558 | 121 | 1.01 (0.99-1.02) | 0.226 |
| BMI (kg/m^2^) | Continuous | 557 | 121 | 1.01 (0.98-1.05) | 0.426 |
|  | <30 vs ≥30 | 382 vs 175 | 76 vs 45 | 0.72 (0.47-1.09) | 0.123 |
| Number of CSEs in the previous 12 months | Continuous | **558** | **121** | **1.21 (1.02-1.44)** | **0.033** |
|  | 0 vs ≥1 | **225 vs 333** | **35 vs 86** | **0.53 (0.34-0.82)** | **0.004** |
|  | ≤2 vs >2 | 497 vs 61 | 104 vs 17 | 0.68 (0.38-1.25) | 0.216 |
|  | ≤3 vs >3 | 540 vs 18 | 115 vs 6 | 0.54 (0.20-1.47) | 0.229 |
| Place of residence | Urban vs Semi-urban/Rural | **382 vs 176** | **70 vs 51** | **0.55 (0.36-0.83)** | **0.005** |
| Sex | Male vs Female | 198 vs 360 | 35 vs 86 | 0.68 (0.44-1.06) | 0.090 |
| Smoking status | Former vs Never | 165 vs 393 | 36 vs 85 | 1.01 (0.65-1.57) | 0.960 |
| Administrative geographic region | Attica vs Outside Attica | **225 vs 333** | **38 vs 83** | **0.61 (0.40-0.94)** | **0.025** |
| Type of institution | Academic vs Other | 222 vs 336 | 47 vs 74 | 0.95 (0.63-1.44) | 0.811 |

The modeled probability was T2-low SA phenotype : 'Yes' vs 'No’.

Abbreviations: BMI, Body Mass Index; CI, Confidence Interval; CSE, Clinically Significant Exacerbation; n_pt_, number of patients; OR, Odds Ratio; SA, Severe Asthma; T2, Type-2.

**Table S4.** Association of the ACT score with factors of interest through univariable logistic regression analysis in the T2-low population.

| **Parameter** | **Category vs Reference** | **n_pt_**  **Overall** | **n_pt_**  **Controlled** | **OR (95% CI)** | **p-value** |
| --- | --- | --- | --- | --- | --- |
| Patient's age at the study visit | Continuous | 121 | 45 | 1.00 (0.97-1.02) | 0.754 |
|  | ≤65 vs >65 | 85 vs 36 | 31 vs 14 | 0.90 (0.40-2.01) | 0.801 |
| Patient's age at asthma symptom onset | Continuous | 121 | 45 | 1.02 (1.00-1.04) | 0.077 |
|  | ≤18 vs >18 | 16 vs 105 | 4 vs 41 | 0.52 (0.16-1.72) | 0.285 |
| Patient's age at SA diagnosis | Continuous | 121 | 45 | 1.00 (0.98-1.02) | 0.915 |
| Number of CSEs in the previous 12 months | Continuous | **121** | **45** | **0.36 (0.22-0.59)** | **<0.001** |
|  | 0 vs ≥1 | **35 vs 86** | **20 vs 25** | **3.25 (1.44-7.35)** | **0.005** |
| Place of residence | Urban vs Semi-urban/Rural | 70 vs 51 | 30 vs 15 | 1.80 (0.84-3.87) | 0.133 |
| Sex | Male vs Female | 35 vs 86 | 12 vs 33 | 0.84 (0.37-1.91) | 0.673 |
| Smoking status | Former vs Never | 36 vs 85 | 12 vs 33 | 0.79 (0.35-1.79) | 0.568 |
| Administrative geographic region | Attica vs Outside Attica | 38 vs 83 | 16 vs 29 | 1.35 (0.62-2.97) | 0.450 |
| Type of institution | Academic vs Other | **47 vs 74** | **24 vs 21** | **2.63 (1.23-5.65)** | **0.013** |
| Employment status | Employed vs Other | 56 vs 65 | 19 vs 26 | 0.77 (0.37-1.62) | 0.491 |
| Alcohol consumption | <2 vs ≥2 units/week | 108 vs 13 | 38 vs 7 | 0.47 (0.15-1.48) | 0.196 |
| Physical activity | Active vs Inactive | 50 vs 71 | 20 vs 25 | 1.23 (0.58-2.59) | 0.592 |
| Comorbidities/risk factors/triggers for asthma symptoms & exacerbations | Yes vs No | 93 vs 28 | 36 vs 9 | 1.33 (0.54-3.27) | 0.529 |
| History of food allergy | Yes vs No | 12 vs 109 | 6 vs 39 | 1.79 (0.54-5.94) | 0.338 |
| Chronic rhinosinusitis and nasal polyposis | Yes vs No | 11 vs 110 | 7 vs 38 | 3.31 (0.91-12.04) | 0.069 |
| Chronic rhinosinusitis | Yes vs No | 38 vs 83 | 16 vs 29 | 1.35 (0.62-2.97) | 0.450 |
| Allergic rhinitis | Yes vs No | 50 vs 71 | 21 vs 24 | 1.42 (0.67-2.99) | 0.359 |
| Nasal polyposis | Yes vs No | **12 vs 109** | **8 vs 37** | **3.89 (1.10-13.77)** | **0.035** |
| Prior nasal surgery | Yes vs No | 9 vs 112 | 4 vs 41 | 1.39 (0.35-5.45) | 0.641 |
| Gastroesophageal Reflux Disease | Yes vs No | 35 vs 86 | 12 vs 33 | 0.84 (0.37-1.91) | 0.673 |
| Environmental tobacco exposure | Yes vs No | 26 vs 95 | 10 vs 35 | 1.07 (0.44-2.62) | 0.880 |
| Adherence to asthma therapy & correct inhaler technique | High vs Other | 95 vs 26 | 35 vs 10 | 0.93 (0.38-2.28) | 0.880 |
| Current receipt of biologic treatment | Yes vs No | 27 vs 94 | 14 vs 31 | 2.19 (0.92-5.22) | 0.077 |
| HADS total score | ≥15 vs <15 | **49 vs 63** | **10 vs 31** | **0.26 (0.11-0.62)** | **0.002** |
| BMI (kg/m^2^) | Continuous | 121 | 45 | 1.00 (0.95-1.06) | 0.901 |
|  | <30 vs ≥30 | 76 vs 45 | 29 vs 16 | 1.12 (0.52-2.41) | 0.775 |
| FEV_1_ | <65% vs ≥65% | **30 vs 91** | **4 vs 41** | **0.19 (0.06-0.58)** | **0.004** |
| BEC (cells/μL) | Continuous | 121 | 45 | 1.00 (0.99-1.00) | 0.569 |
|  | <150 vs ≥150 | 96 vs 25 | 35 vs 10 | 0.86 (0.35-2.12) | 0.744 |
| IgE (IU/mL) | Continuous | 119 | 45 | 1.00 (1.00-1.00) | 0.684 |
|  | <100 vs ≥100 IU/mL | 92 vs 27 | 33 vs 12 | 0.70 (0.29-1.67) | 0.420 |

The modeled probability was 'ACT≥20' versus 'ACT<20’. The following covariates were not included due to quasi/complete separation issues and/or extremely unbalanced groups: CSEs in the previous 12 months (≤2 vs >2; ≤3 vs >3) and current mOCS (Yes vs No).

Abbreviations: ACT, Asthma Control Test; BEC, Blood Eosinophil Count; BMI, Body Mass Index; CI, Confidence Interval; CSE, Clinically Significant Exacerbation; FEV_1_, Forced-Expiratory Volume in 1 second; HADS, Hospital Anxiety and Depression Scale; IgE, Immunoglobulin E; mOCS, maintenance Oral Corticosteroids; n_pt_, number of patients; OR, Odds Ratio; SA, Severe Asthma; T2, Type-2.

**Figure S1.** Overview of study design.

*≥1 IgE measurement before omalizumab initiation for omalizumab-treated patients.

Abbreviations: ACT, Asthma Control Test; AQLQ, Asthma Quality of Life Questionnaire; BEC, Blood Eosinophil Count; FeNO, Fractional exhaled Nitric Oxide; HADS, Hospital Anxiety and Depression Scale; HCRU, Healthcare Resource Utilization; IgE, Immunoglobulin E; mOCS, maintenance Oral Corticosteroids; PROs, patient-reported outcomes; RAST, Radioallergosorbent Test; SA, Severe Asthma; SPT, Skin Prick Test; T2, Type-2; WPAI:RS, Work Productivity and Activity Impairment:Respiratory Symptoms.

**Figure S2.** Frequency of possible and definite T2-low and T2-high SA categories, per phenotype definition (BASE or STRICT), in the overall population and per current receipt of biologic and/or OCS treatment.

Abbreviations: mOCS, maintenance Oral Corticosteroids; n, number of patients with variable; N, number of patients with available data; SA, Severe Asthma; T2, Type-2.

**Figure S3.** Information on biomarkers at most recent measurement before study visit, in the overall T2-low SA population, its subpopulations by ACT-based asthma control level, and the T2-high SA population.

*27 patients had no measurement in the past 12 months or at the study visit.

Box-plots depict median with IQR, including whiskers that extend from minimum to maximum values.

Abbreviations: ACT, Asthma Control Test; BEC, Blood Eosinophil Count; FeNO, Fractional exhaled Nitric Oxide; IgE, Immunoglobulin E; IQR, Interquartile Range; N, number of patients with available data; SD, Standard Deviation; T2, Type-2; Uncontr., Uncontrolled.

**Figure S4.** (A) Asthma-specific functional impairments and (B-C) hospital anxiety and depression at the study visit, in the overall T2-low SA and its subpopulations by ACT-based asthma control level.

Box-plots depict median with IQR, including whiskers that extend from minimum to maximum values.

Abbreviations: ACT, Asthma Control Test; AQLQ, Asthma Quality of Life Questionnaire; Cont., Controlled; HADS-T, Hospital Anxiety and Depression Scale-Total score; IQR, Interquartile Range; N, number of patients with available data; SA, Severe Asthma; SD, Standard Deviation; T2, Type-2; Uncontr., Uncontrolled.

**Figure S5.** (A) Work productivity and activity impairment at the study visit, and (B) asthma-related HCRU in the 12 months before study visit, in the overall T2-low SA and its subpopulations by ACT-based asthma control level.

*Number of unscheduled visits was unknown for one patient.

Box-plots depict median with IQR, including whiskers that extend from minimum to maximum values.

Abbreviations: ACT, Asthma Control Test; HCRU, Healthcare Resource Utilization; IQR, Interquartile Range; N, number of patients with available data; n_pt_, number of patients; SA, Severe Asthma; SD, Standard Deviation; T2, Type-2; WPAI:RS, Work Productivity and Activity Impairment: Respiratory Symptoms.
